# Regulatory effect of Pseudomonas aeruginosa mannose-sensitive hemagglutinin on inflammation and immune function in percutaneous nephrolithotomy patients with upper urinary tract calculi complicated with infection

**DOI:** 10.3389/fimmu.2023.1181688

**Published:** 2023-06-12

**Authors:** Yuan Zhao, Yafei Zhang, Jianhe Liu

**Affiliations:** ^1^ The Department of Urology, The Second Affiliated Hospital of Kunming Medical University, Kunming, China; ^2^ The Department of Urology, Kunming First People’s Hospital: Affiliated Calmette Hospital of Kunming Medical University, Kunming, China

**Keywords:** inflammation, percutaneous nephrolithotomy (PCNL), lymphocyte subsets, immunomodulator, Pseudomonas aeruginosa mannose-sensitive hemagglutinin

## Abstract

**Objective:**

To study the effect of an injection of Pseudomonas aeruginosa mannose-sensitive hemagglutinin (PA-MSHA) on inflammation and immune function in patients with upper urinary tract calculi complicated by infection who have undergone percutaneous nephrolithotomy.

**Methods:**

We retrospectively recorded the clinical data of patients with upper urinary tract calculi complicated by infection who have undergone Percutaneous nephrolithotomy(PCNL) in the Department of Urology, 2nd Affiliation Hospital of Kunming Medical University, from March to December 2021. Clinical data include general condition, laboratory index, CT, postoperative body temperature, heart rate, respiration, SIRS, sepsis, etc. Patients were divided into treated and control groups according to whether they had received a preoperative PA-MSHA injection. The two groups were compared for indices of inflammation and complications of infection after PCNL. Pre- and post-operative lymphocyte subsets and immunoglobulin changes were compared.

**Results:**

115 patients were included in the study, including 43 in the treatment group and 72 in the control group. After Propensity Score Matching, 90 patients were divided into treatment (n=35) and control (n=55) groups. The postoperative inflammation index was higher in the treatment group than in the control group (P<0.05). The incidence of postoperative SIRS was higher in the treatment group than control (P<0.05). There were no cases of sepsis in either group. The double-positive T cells lymphocyte subsets were higher in the treatment group than in the control group ((P<0.05). Pre- and post-operative changes in immune function: total T lymphocyte count reduced, NK and NKT cell count increased in the control group, double-positive T cell count increased in the treatment group, IgG, IgA, IgM, complement C3 and C4 count reduced in both groups post-operatively.

**Conclusion:**

This study found that patients with upper urinary tract calculi and infection treated with antibiotic-based PA-MSHA before percutaneous nephrolithotomy had an increased inflammatory response after surgery, which may play a role in the prevention and treatment of sepsis. The percentage of double-positive T cells in the peripheral blood was increased after PA-MSHA treatment, which may have an immunomodulatory and protective effect in PCNL patients with stones complicated by infection.

## 1 Introduction

A recent study found that age-standardized morbidity and mortality from sepsis have decreased over the past 30 years; however, 19.7% of deaths worldwide are still attributed to sepsis (1). Even in high-income countries, case fatality rates for sepsis and severe sepsis are as high as 17% and 26%, respectively, making it a leading cause of morbidity and mortality worldwide (2). Percutaneous nephrolithotomy is associated with less trauma, a high stone extraction rate and rapid postoperative recovery and is a first-line treatment for high-volume upper urinary tract calculi. Currently, the only preventive measure available for urinary sepsis after PCNL are limited to preoperative antibiotic prophylaxis was used routinely for 7d for the high-risk population, nephrostomy and other measures to relieve obstruction in time before surgery (3, 4), maintaining a low perfusion pressure during surgery, controlling the duration of surgery and keeping the drainage unobstructed after surgery (5). The prevention of urinary sepsis, which can occur after surgery, has always been the difficulty and focus for urologists to improve the perioperative safety of patients undergoing endoscopic surgery (6).

Sepsis can trigger complex immune interactions between pro-inflammatory and anti-inflammatory in the host. During sepsis, inflammatory storms cause damage to the body, meantime immune activation and inflammation are needed to fight infection, and blocking inflammation can be fatal. Considering its complex immune response, the similarity between immunosuppression and immunodeficiency of cancer (7), and the increasing incidence of sepsis (8). At the same time, the current treatment is still limited to the general treatment of ICU (9) and the gradual emergence and development of antibiotic resistance. These all provide a driving force for developing immune regulation and immunostimulatory therapy for sepsis. Exploring and developing treatments to improve the dynamic balance of the immune system is a new way to treat sepsis, a disease of immune imbalance. The strategies of immune regulation and immunostimulatory therapy show excellent prospects in clinical research (9).

Immunotherapy includes passive immunization, active immunization, and other immunoadjuvant therapy. In passive immunity, the body acquires immunity passively based on the antibacterial activity of antibodies (neutralizing toxin, blocking pathogen invasion, activating complement, leading to phagocytosis, cytotoxicity) and immunomodulatory activity (down-regulating the production of inflammatory cytokines IL-1, IL-2, TNF α, IGF, accelerating antibody clearance) (8). Injection of specific pathogenic bacteria antigens to improve the body’s resistance to the pathogenic bacteria infection is called active immunity. Active immune candidate vaccine components for sepsis include endotoxin, bacterial superantigen, peptidoglycan, teichoic acid, bacterial DNA, and other microbial mediators (8). Vaccine methods can complement standard treatment schemes and other immunotherapy measures. It may be an effective way to prevent sepsis by immunizing people at high risk of sepsis to produce high titers of protective antibodies against common harmful bacteria and toxins. Among them, PA-MSHA injection is a biological vaccine preparation with trans-bacterial immunogenicity, and its main component is Pseudomonas aeruginosa mannose-sensitive hemagglutination hair strain. A large number of basic medical studies, clinical applications, and studies for many years have shown that PA-MSHA injection can induce the activation of dendritic cells, activate the adaptive immune response, activate macrophages, improve tumor microenvironment, specifically adhere to tumor cells, block EGFR signal pathway and induce tumor cell apoptosis. Clinically, it is used to treat malignant tumors, regulate and improve human immune function, and reduce the occurrence of infection (10–12).

By detecting the changes in T lymphocytes and immunoglobulin, we can understand the autoimmune status of patients, then effectively guide clinical treatment. T lymphocytes of different types and subsets assume their responsibilities to achieve the functions of immune activation, response, promotion, or inhibition of immune response. When T cells are dysfunctional, it can lead to autoimmunity or other diseases. For example, there is a T lymphocyte immune disorder in sepsis (13). Thus, the aim of this study was to understand the changes in immune function before and after PCNL, and more importantly, to detect and compare the lymphocytic subsets, immunoglobulins, inflammation indices, and infection complications (SIRS, sepsis) in infected patients through the detection of lymphocytic subsets and immunoglobulins. To further discuss the regulatory effect of PA-MSHA injection on immune function and inflammation in patients with calculi complicated by infection undergoing PCNL surgery.

## 2 Materials and methods

### 2.1 Clinical data of PCNL patients with upper urinary tract calculi and infection in the Department of Urology of the second affiliated Hospital of Kunming Medical University from March to December 2021

#### 2.1.1 Inclusion criteria

(1) age: 18-80;
(2) patients underwent percutaneous nephrolithotomy for upper urinary tract calculi;
(3) meet the diagnostic criteria of urinary tract infection.

#### 2.1.2 Exclusion criteria

(1) patients with other malignant tumors;
(2) patients treated with immunosuppressants;
(3) patients with immunodeficiency disease;
(4) pregnant or lactating women;
(5) patients with incomplete clinical data collection.

### 2.2 Grouping method

The patients were divided into treatment group and control group according to whether they received PA-MSHA injection before operation.

### 2.3 Treatment scheme

#### 2.3.1 Antibacterial therapy

According to the HALF classification, the high-risk and asymptomatic bacteriuria groups were treated with sensitive antibiotics according to urine culture results before the operation; if the urine culture was negative, antibiotics covering common urinary tract pathogens were used for 1 week. In the low-risk group, the first-and second-generation cephalosporins and fluoroquinolones were selected; if there were no postoperative complications, the total course of antibiotics was less than 24 hours. In the fever group, infection was controlled in the first stage, second expectation of infection symptoms and management of stones once infection indicators have stabilized; the antibiotics in the perioperative period were selected according to the positive medication sensitivity results of the latest urine or blood bacteriological culture, and the main route of administration was intravenous infusion.

H group, high risk: Preoperative negative urine culture, patients without febrile symptoms, but with high risk factors for intraoperative and postoperative infectious complications, including large stone load (≥2 cm in diameter) and/or moderate or severe hydronephrosis, recent history of fever, presence of urinary routine infection, long-term indwelling catheter, diabetic patients, and immunocompromised patients. A group, asymptomatic bacteriuria: Positive preoperative urine culture or preoperative urine routine suggesting positive nitrite, but the patient has no symptoms of infection. L group, low risk: Preoperative urine culture was negative, the patient had no chills or fever, stone diameter <2 cm, no obstruction or incomplete obstruction, and no or mild hydronephrosis. F group, fever: Patients with urinary stones combined with obstruction present with symptoms of urinary tract infection such as chills and fever.

#### 2.3.2 Pseudomonas aeruginosa mannose-sensitive hemagglutinin treatment

PA-MSHA: Chinese medicine standard word S20043022, specification: containing bacteria 1.8x109/ml, Beijing Wante Biopharmaceutical Co., Ltd. It is suitable for adjuvant treatment of malignant tumors, improving the human immune status and reducing the occurrence of infection. Adverse reactions: local injection site has mild redness and swelling, very few have a low fever, do not need to deal with can be self-subsiding.

The patients in the treatment group were subcutaneously injected with PA-MSHA injection; the injection site was at the upper arm or around the umbilical cord, the injection dose was 1ml/per time, the frequency was once every other day, and the injection was completed three times before PCNL.

#### 2.3.3 Anesthesia method

Intravenous inhalation combined with anesthesia was used. The induction regimen was propofol-induced 2mg/kg, sufentanyl 0.4ug/kg, and rocuronium 0.9mg/kg. The maintenance dose was propofol 4mg/kg/h, remifentanil 0.15ug/kg/min. Sevoflurane inhalation maintained the MAC at 0.7.

#### 2.3.4 Percutaneous nephrolithotomy

The operation on the patient was performed by physicians with at least 2 years of experience in percutaneous nephrolithotomy. After the anesthesia took effect, they found the lithotomy position of the patient, routinely disinfected the perineal skin, and spread aseptic disinfection towels. Ureteroscope was inserted through the urethra, and a ureteral catheter was inserted along the ureteral orifice of the affected side. The depth of the ureteral catheter was about 25cm. After urine outflow, the ureter was retained, and the ureteral catheter was fixed. The patient took the prone position, sterilized the skin of the operation area, and spread the aseptic operation towel. Artificial hydronephrosis was formed by injecting normal saline through the ureteral catheter. The puncture point was selected at the suprascapular line of the operation side and the upper/lower ribs. Under the guidance of ultrasound/X-ray, the puncture into the target calyx and the urine outflow was seen by the puncture needle. The guide wire was inserted into the puncture needle, the fascia dilatation tube was used to expand to F20 step by step, the F20 working sheath was retained, and the ureteroscope/nephroscope was placed. The renal pelvis and calyceal structure, color, and shape were observed. The stones were smashed one by one with a holmium laser and sucked out and collected until there was no residual stone after repeated observation. Under the guidance of the guide wire, one F5 double J tube was inserted, withdrew from the nephroscope, and one F16 nephrostomy tube was appropriately fixed at the end of the operation.

### 2.4 Clinical data

#### 2.4.1 Demographic and clinical characteristics of patients

(1) Sex, age, BMI;
(2) The maximum diameter of upper urinary tract calculi measured by computerized tomography in the middleand lower abdomen;
(3) Urine leukocyte count, urine culture, IL-6, hs-CRP, PCT, peripheral blood leukocyte count, serum creatinine value;
(4) Days of preoperative use of antibiotics.

#### 2.4.2 Related indicators of postoperative inflammatory response

(1) IL-6, hs-CRP, PCT;
(2) SIRS related index: peripheral blood leukocyte count, proportion of peripheral blood immature cells, arterial blood carbon dioxide partial pressure, heart rate, body temperature, respiratory rate;
(3) Sepsis related index: PO2/FiO2, platelet, bilirubin, mean arterial pressure, vasoactive medication use, creatinine, urine volume, central nervous system score.

Definitions of sepsis(2016,sepsis-3): Suspected infection and an acute change in Sequential Organ Failure Assessment (SOFA) score of ≥2 points consequent to infection.

#### 2.4.3 Related indicators of immune function

(1) Lymphocyte subsets detection: total T lymphocyte percentage, T4 lymphocyte percentage, T8 lymphocyte percentage, double negative T lymphocyte percentage, CD4+CD8+double positive T lymphocyte percentage, B lymphocyte percentage, NKT lymphocyte percentage, NK lymphocyte percentage, HLA-DR+T lymphocyte percentage, CD4/CD8 ratio.

(2) five items of immunoglobulin: IgG, IgA, IgM, complement C3, complement C4.

### 2.5 Research methods

Clinical data were collected retrospectively, the inflammatory indexes, infection complications [SIRS, sepsis (14)], lymphocyte subsets, and immunoglobulin before and after PCNL were compared between the two groups.

### 2.6 Statistical methods

SPSS26.0 software was used to process, analyze, and count the data.

The baseline of the two groups of original data needs to be balanced. Propensity score matching (PSM) was used, with a matching ratio of 1:2, so that study groups were similar to those in randomised controlled trials. The adoption rate or constituent ratio of counting data was expressed, and the chi-square or Fisher’s accurate test was used for comparison. If the data were normally distributed, the mean ± standard deviation (SD), independent samples t-test, and paired t-test were applied to compare between groups. If the data are not normally distributed, use the interquartile range (IQR) to compare the differences.

## 3 Result

### 3.1 demographic and clinical characteristics

In this study, 115 patients were divided into treatment (n=42) and control (n=73) groups. There was no significant difference in age, sex, BMI, urine leukocyte count, urine culture positive rate, blood leukocyte count, PCT, hs-CRP, creatinine, and days of preoperative antibiotic treatment between the two groups. However, there was a significant difference in stone diameter and IL-6 between the two groups, as shown in **Table 1**.

**Table 1:** Demographics and clinical characteristics.

		Control group	Treatment group	
Project		(n=73)	(n=42)	P Value
age(yr),mean ± SD		47.97 ± 10.69	50.64 ± 7.72	0.159
gender (%)	woman	23(31.5)	20(47.6)	0.129
	man	50(68.5)	22(52.4)	
BMI,mean ± SD		24.09 ± 3.57	24.34 ± 3.36	0.711
stone diameter(cm),median [IQR]		1.8 [1.5, 2.7]	2.65[1.97,3.10]	0.003
creatinine(μmoI/L),median [IQR]		85.0[75.0, 100.0]	81.0[69.0, 94.0]	0.135
Urine leukocyte count without antimicrobial therapy(/uL),median 7[IQR]		210.3[112.8, 673.2]	274.3[125.85,1157.62]	0.111
positive urine cultures(%)		17(23.3)	14(33.3)	0.242
numbers of white blood cells(×10^9^/L),median [IQR]		6.51[5.65,7.89]	6.845[5.77,8.1425]	0.296
IL-6(pg/ml), median [IQR]		2.58[1.49, 8.5]	6.2[2.6775, 9.775]	0.036
PCT(ng/ml),median [IQR]		0.04[0.03,0.06]	0.045[0.03,0.0675]	0.526
hs-CRP(mg/L),median [IQR]		2.51[1.11,8.7]	4.455[2.315,12.5525]	0.156
Days of preoperative antimicrobial therapy(d),median [IQR]		3[2,5]	4[3,6]	0.160

Of the 42 patients in the treatment group, 14 patients (33.3%) had pain at the injection site of the PA-MSHA injection, and 8 cases (19%) had redness and swelling at the injection site at the same time. All patients’ pain, redness and swelling resolved without any special treatment.

According to PSM matching, there were 35 cases in the treated group and 55 in the control group. See **Tables 2**, **3** for details.

**Table 2:** Demographic and clinical characteristics after PSM matching.

		Control group	Treatment group	
Project		(n=55)	(n=35)	P value
age(yr),mean ± SD		50.09 ± 9.49	50.57 ± 7.4	0.8
gender (%)	Woman	18 (32.7)	14 (40.0)	0.634
	Man	37 (67.3)	21 (60.0)	
BMI,mean ± SD		23.97 ± 3.45	24.21 ± 2.99	0.735
stone diameter (cm),median [IQR]		2.2 [1.6,2.9]	2.3 [1.8,2.96]	0.303
Urine leukocyte count without antimicrobial therapy (/uL),median [IQR]		238.4 [124.2,742.15]	239.7 [119.5,801.15]	0.888
positive urine cultures (%)		16(29.1)	11(31.4)	0.813
numbers of white blood cells (×10^9^/L),median [IQR]		6.48[5.62,7.645]	6.81[5.705,7.85]	0.495
IL-6(pg/ml), median [IQR]		2.58[1.49,6.545]	5.11[2.41,6.97]	0.076
PCT(ng/ml),median [IQR]		0.04[0.025,0.06]	0.04[0.025,0.06]	0.706
hs-CRP(mg/L),median [IQR]		3.53[1.095,8.43]	4.23[1.79,9.855]	0.387
creatinine (μmoI/L),median [IQR]		85.0 [74.5,105.5]	82.0 [72.0,101.0]	0.376
Days of preoperative antimicrobial therapy (d),median [IQR]		3[2.5,5]	4[3,5.5]	0.215

**Table 3:** P-values for intra-group comparison and inter-group comparison of lymphocyte subpopulations and immunoglobulin five.

	pre-OP vs. post-OP		Treatment group vs control group	
	Control group	Treatment group	Pre-operative	Post-operative
CD3+T	0.013^*^	0.226	0.277	0.369
CD4+T	0.099	0.434	0.269	0.835
CD8+T	0.099	0.487	0.764	0.714
DNT	0.491	0.459	0.691	0.646
CD4+CD8+DPT	0.305	0.017^*^	0.083	0.013^*^
B	0.175	0.451	0.464	0.487
NKT	0.048^*^	0.504	0.910	0.509
NK	0.011^*^	0.516	0.075	0.245
HLA-DR+T	0.272	0.417	0.474	0.924
CD4+/CD8+	0.541	0.600	0.637	0.947
IgG	0.000^*^	0.000^*^	0.446	0.961
IgA	0.000^*^	0.000^*^	0.066	0.067
IgM	0.000^*^	0.000^*^	0.369	0.396
complement C3	0.000^*^	0.000^*^	0.841	0.236
complement C4	0.000^*^	0.000^*^	0.236	0.071

*The difference between each group was statistically significant (P<0.05).

### 3.2 Indexes of postoperative inflammation and occurrence of infection complications (SIRS, sepsis)

After surgery, there were significant differences between the two groups regarding SIRS% and hs-CRP. SIRS was 10.9% (6 cases) in the control group and 31.4% (11 cases) in the treatment group. There were no cases of sepsis in either of the two groups. As shown in **Tables 4**, **5**, there was no statistical difference in IL-6 and PCT between the two groups after surgery.

**Table 4:** Comparison of inflammatory indicators and occurrence of infectious complications (SIRS, sepsis) between groups after PSM matching.

24 h after surgery	Control group (n=55)	Treatment group (n=35)	P value
IL-6(pg/ml), median [IQR]	20.25[13.805,45.49]	26.37[17.955,85.59]	0.077
PCT(ng/ml),median [IQR]	0.05[0.03,0.07]	0.05[0.04,0.065]	0.643
hs-CRP(mg/L),median [IQR]	1.5[0.785, 4.075]	4.54[2.695, 12.4]	0.000
SIRS(%)	6(10.9)	11(31.4)	0.015
Sepsis (%)	0	0	–

**Table 5:** Number of postoperative SIRS cases meeting each diagnostic criterion specifically.

	Control group n=6	Treatment group n=11
Heart rate> 90 counts/minute	6	9
Body temperature>38°Cor < 36°C	4	9
PaCO_2_< 32 mmHg or respiration rate> 20 counts/minute	4	3
Peripheral Blood Leukocyte Count>12×10^9^/L or< 4×10^9^/L Or immature cells≥ 10%	1	6

### 3.3 Lymphocyte subsets and immunoglobulin

#### 3.3.1 Intra-group comparison

**Figure 1:**
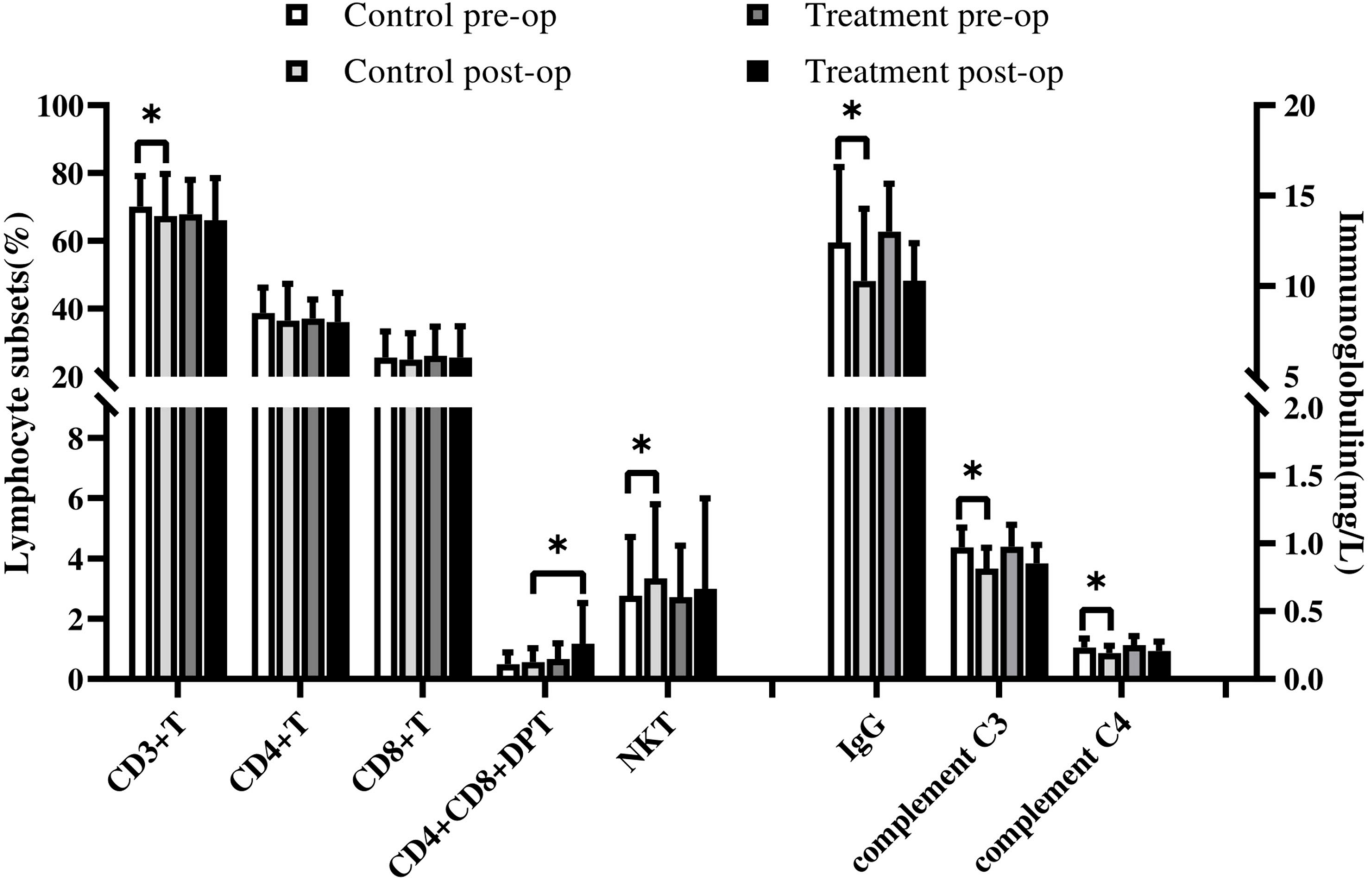
In the control group, NK lymphocyte percentages increased postoperatively. In contrast, IgA, IgM decreased significantly postoperatively. Symbols indicate a significant difference between the indicated groups, as follows: *p < 0.05.

**Figure 2:**
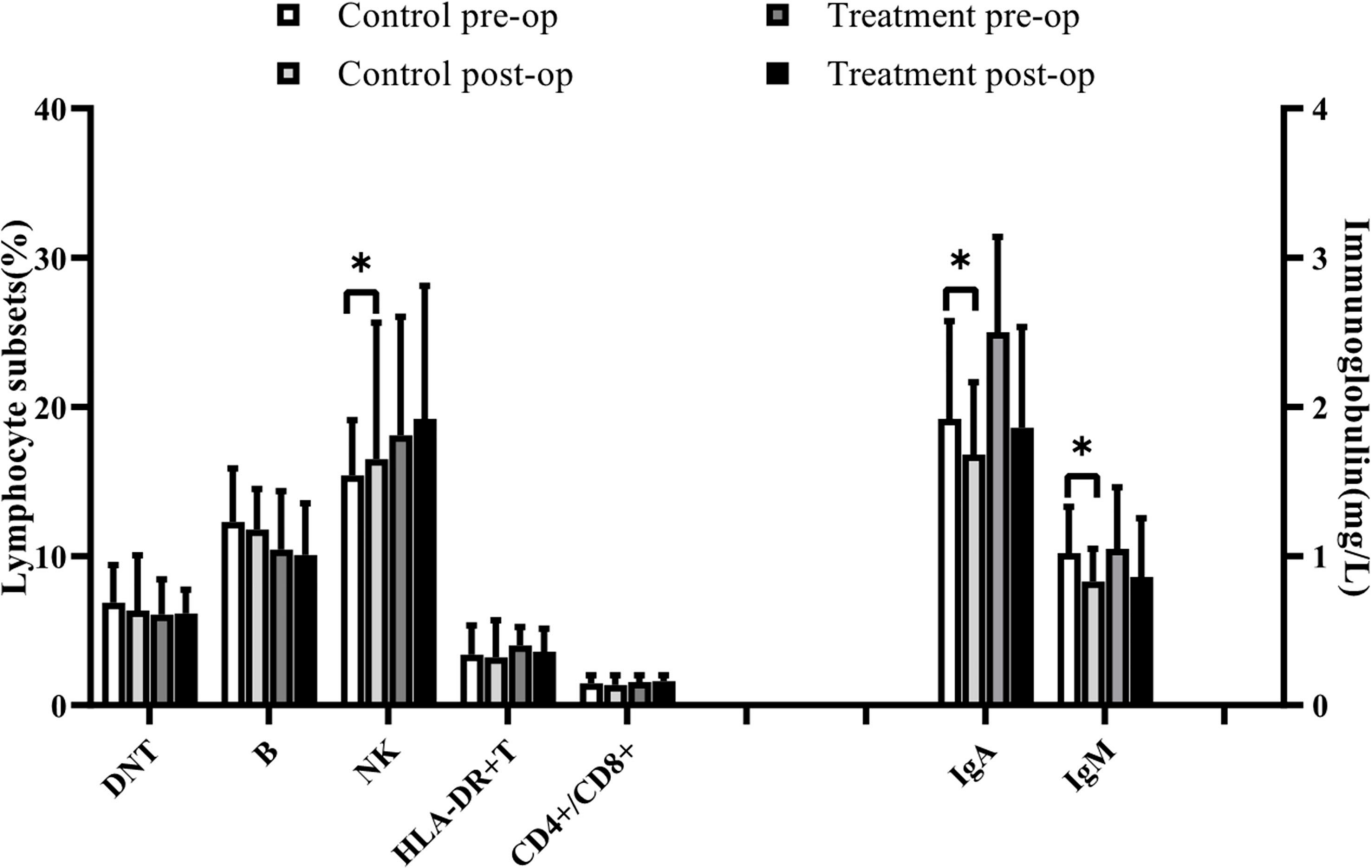
In the control group, NKT lymphocyte percentages increased postoperatively. In contrast, CD3 +T lymphocyte, IgG, complement C3, and complement C4 decreased significantly postoperatively. Comparison between groups: After surgery, CD4+CD8+DPT cell percentages were higher in the treatment group than in the control group. Symbols indicate a significant difference between the indicated groups, as follows: *p < 0.05.

In the control group, NKT and NK lymphocyte percentages increased postoperatively. In contrast, CD3 + T lymphocyte, IgG, IgA, IgM, complement C3, and complement C4 decreased significantly postoperatively, and the other indices were not statistically different, as shown in **Tables 3**, **6** (**Figures 1**, **2**).

**Table 6:** Specific statistical values for lymphocyte subsets and immunoglobulins in the control and treatment groups, preoperatively and postoperatively.

	Control group		Treatment group	
	Pre-operative	Post-operative	Pre-operative	Post-operative
CD3+T	70.1147 ± 9.04614	67.3339 ± 12.44194	67.8657 ± 10.18144	66.099 ± 12.39672
CD4+T	38.7382 ± 7.46683	36.4836 ± 10.82307	37.1030 ± 5.57091	36.0314 ± 8.64461
CD8+T	25.5782 ± 7.72992	24.9582 ± 7.80998	26.1057 ± 8.61998	25.6229 ± 9.20195
DNT	6.88[4.445,9.39]	6.36[4.6,10.045]	6.08[4.655,8.435]	6.17[5.08,7.765]
CD4+CD8+DPT	0.4885 ± 0.39375	0.5625 ± 0.45954	0.6673 ± 0.51074	1.1755 ± 1.34047
B	12.27[7.535,15.875]	11.78[7.38,14.495]	10.43[8.125,14.355]	10.08[7.16,13.55]
NKT	2.7671 ± 1.94983	3.3378 ± 2.45798	2.7217 ± 1.70340	2.9969 ± 2.9969
NK	15.4 [11.35,19.1]	16.5[12,25.65]	18.1[14.9,26.05]	19.2[15.5,28.1]
HLA-DR+T	3.4[2.45,5.35]	3.2[1.75,5.7]	4[2.85,5.25]	3.6[1.9,5.15]
CD4+/CD8+	1.47[1.23,2.02]	1.36[1.085,2.015]	1.57[1.115,2.01]	1.6[1.015,2.03]
IgG	12.4100 ± 4.18440	10.2713 ± 3.99802	13.0169 ± 2.64993	10.3074 ± 2.06988
IgA	1.92[1.55,2.575]	1.68[1.28,2.165]	2.5[1.945,3.14]	1.86[1.42,2.535]
IgM	1.02[0.705,1.33]	0.83[0.645,1.05]	1.05[0.76,1.46]	0.86[0.655,1.255]
complement C3	0.97 ± 0.14625	0.8153 ± 0.15302	0.9766 ± 0.15939	0.8531 ± 0.13554
complement C4	0.2333 ± 0.06605	0.1918 ± 0.05348	0.2503 ± .06573	0.206 ± .06852

The units of items in the lymphocyte subpopulation test are % except for CD4+/CD8+; the units of the five immunoglobulin items are mg/L except for IgG units which are g/L.

In the treatment group, the percentage of CD4+CD8+DPT increased postoperatively, whereas IgG, IgA, IgM, complement C3, and complement C4 decreased compared with the preoperative values, and the difference was statistically significant. As shown in **Tables 3**, **6** (**Figures 1**, **2**), the other indices had no statistical difference.

#### 3.3.2 comparison between groups

Lymphocyte subsets and immunoglobulins were not significantly different between the two groups before surgery.

After surgery, CD4+CD8+DPT cell percentages were higher in the treatment group than in the control group, and the difference was statistically significant, with no statistical difference in other indices, as detailed in **Tables 3**, **6** (**Figures 1**, **2**).

## 4 Discussion

Sepsis is a life-threatening organ dysfunction caused by a defective host response to infection (15). Urinary sepsis is caused by an infection of the genitourinary tract (14). It accounts for 9-31% of sepsis cases, with a mortality rate of 20-40% (16). Urinary sepsis is a serious and life-threatening complication of UTI. It is important to recognize it early and start appropriate treatment promptly (17).

### 4.1 Changes of immune function in sepsis

In patients with sepsis, there is a direct relationship between the occurrence and degree of immune dysfunction and adverse outcomes. There are two stages in the immune process of sepsis: immune-activated and immunosuppressed. Initially, a “cytokine storm” mediates excessive inflammation, upregulating innate immunity gene expression while downregulating adaptive immunity gene expression. The body then activates anti-inflammatory mechanisms, reducing immune function and endogenous anti-inflammatory responses, increasing susceptibility to infection (18). Sepsis involves apoptosis, endotoxin tolerance, epigenetic reprogramming and central regulation (19), and up-regulated expression of immunosuppressive molecules (20), including programmed death receptor-1 (Programmed cell Death protein1, PD-1), programmed death ligand-1 (Programmed cell Death 1 Ligand 1, PD-L1), cytotoxic T lymphocyte antigen-4, T cell membrane protein-3 and lymphocyte activating gene-3 directly or indirectly damage the function of almost all types of immune cells, and the immune cells regulate each other, damaging the innate immune system and acquired immune system function (20).

In innate immunity, there is a decrease in the number of dendritic cells, NK cells, and γ δ T cells (21–23). Of these, dendritic cells are susceptible to sepsis (24), with reduced expression (25), reduced antigen presentation, and apoptosis strongly associated with sepsis-associated immunosuppression and death (26). It affects adaptive immunity by inducing T cell anergy and Treg cell proliferation. It does not induce effector T-cell responses (25). Decreased cytokines secreted by NK cells, decreased cytotoxicity (22), and decreased IFN-γ produced by NK cells also lead to decreased HLA-DR expression on monocytes, promoting immunosuppression (27). The reactivation of latent viruses may also result from impaired NK cell function (27). Monocyte-macrophage cells showed reduced ability to release proinflammatory cytokines but unimpaired or increased ability to release anti-inflammatory mediators. Antigen presentation was reduced due to the increased release of immunosuppressive mediators, HLA-DR expression was also reduced (22). After ingestion of apoptotic immune cells by surviving monocytes-macrophages and dendritic cells, a state of “immune tolerance” with characteristics of immunosuppression occurred, antigen presentation function decreased, and its function was insufficient to fight pathogens (18, 21). And neutrophils, which account for the highest proportion of leukocytes, although the number of neutrophils increased in circulation, but their function decreased (28).

Adaptive immunity involves a reduction of cytokine production by the Th 1, Th 2 and Th 17 cell subsets of CD4+ Th cells. Cytokines produced by Th1, Th2 and Th17 cell subsets (IFN-γ, IL-2, TNF-α, IL-4, IL-5, IL-6, IL-17, etc.) play an essential role in normal cellular immunity, humoral immunity and innate immunity (29). Expression of PD1 in CD4+ T cells and PDL1 on macrophages increased, and PDL1 expression in capillary endothelial and bronchial epithelial cells increased. In this case, even if T cells have migrated to the site of infection, their function is impaired, severely compromising the host’s ability to scavenge microorganisms (18). Studies have shown that increased PD1 expression on circulating T cells in patients with sepsis is associated with decreased T cell proliferation, increased nosocomial infection and mortality (30). Treg cells are a specific group of T cells and “inhibitory” cells. In sepsis, Treg cells are more resistant to sepsis-induced apoptosis and increase in number. Treg cells weaken innate and acquired immunity by inhibiting monocytes, neutrophils, NK cells and effector T cells, further increasing susceptibility to secondary microbial infection (31, 32).

### 4.2 Immunotherapy for immunosuppression

For immunosuppression, effective immunotherapies include intravenous immunoglobulin (33), IFN γ (34), GM-CSF (19), recombinant human IL-7 (35), thymosin α1 (36), anti-PD-1 and anti-PDL-1 (37), etc. J5DLPS/OMP composite vaccine (38), chip vaccine (39), IL-3 (40), IL-15 (41), FLT3L (42), PA-MSHA (11), etc., require further experimental investigation.

PA-MSHA injection, the vaccine is an inactivated Pseudomonas aeruginosa mannose-sensitive haemagglutinin fimbriae mutant that contain lipopolysaccharide antigen in the outer membrane of the bacterial cell wall and Pseudomonas aeruginosa MSHA fimbriae. The main component of the pilus is the highly immunogenic fimbrial protein, which is widely distributed in Gram-negative bacilli of the same species, such as Escherichia coli, Proteus, etc. The vaccine, therefore, has broad-spectrum immunogenicity. The activation of immune cells can produce a high titer of broad-spectrum pili antibodies (the main component is IgG) and improve the activity of NK cells, the level of IL-2, complement C3, complement C4, and the CD4/CD8 ratio (43). Currently, it has been shown to improve immune function in malignant tumors such as bladder, lung, breast, and skin cancers and chronic idiopathic urticaria (44–49). In terms of inflammatory regulation, the vaccine has both pro- and anti-inflammatory effects.

PA-MSHA can activate dendritic cell proliferation and differentiation, alter immune tolerance, activate T and B cells, increase T lymphocyte numbers and subsets and proportion of CD4+T lymphocytes, stimulate differentiation of innate immune factors, and improve antigen presentation function (11). Liu, X.F. et al. analysed changes in cellular immunity and inflammatory factors of PA-MSHA and septic serum-stimulated healthy human peripheral blood mononuclear cells (PBMC) and found that PA-MSHA pretreatment promoted the production of Tregs cells, increased the level of IL-10 and inhibited the production of TNF-α induced by septic serum (50).

### 4.3 PA-MSHA in patients with upper urinary tract calculi complicated with infection

#### 4.3.1 Inflammatory indicators

The prevention and treatment of sepsis after percutaneous nephrolithotomy in patients with upper urinary tract calculi complicated with infection is a major challenge. Given the potential regulation of inflammatory response shown by the PA-MSHA vaccine, we studied its role in the early prevention of sepsis. The postoperative inflammation index and the incidence of infection complications were statistically analyzed and compared, and it was found that there was no significant difference between PCT and IL-6. The hs-CRP of the treatment group was higher than that of the control group, and the difference was statistically significant. However, the median of the treatment group was still within the normal reference range. Hs-CRP is an acute phase reactive protein significantly increased in infection, which can activate monocytes-macrophages, etc., and is an essential inflammatory factor promoting downstream inflammatory response (51). This study found that the incidence of SIRS in the treatment group was higher than in the control group, and the inflammatory reaction was more robust. However, according to the criteria of SOFA and qSOFA, there was no sepsis in the two groups. Systemic inflammatory response syndrome is a group of clinical symptoms of the systemic non-specific inflammatory response caused by a severe injury of infectious or non-infectious factors such as infection, trauma, burn, operation, and ischemia-reperfusion (52). The indexes in SIRS criteria, such as body temperature, white blood cells, and respiration, are more basic indicators that reflect the host’s response more than the disorder’s response (52). In other studies, the incidence of SIRS after PCNL was 20-30%, and sepsis was only 0-3% (53). Compared with SIRS, SOFA, and qSOFA, the sensitivity and specificity are better than SIRS, which can better reflect the homeostasis imbalance in the body. SOFA score and qSOFA score can assess the risk of sepsis in infected patients and greatly help in screening suspected sepsis (54). Considered the incidence of SIRS in the treatment group is higher than that in the control group, and the increased inflammatory reaction is related to the medication reaction.

This is similar to the results observed by Zhu, H et al. (55) in a septic mouse model. In this study, the levels of pro-inflammatory factors (TNF- α and IL-6) were significantly increased in rats pretreated with PA-MSHA every other day for 8 days; on the contrary, the levels of anti-inflammatory factors (IL-4 and IL-10) increased, and the levels of pro-inflammatory factors (TNF- α and IL-6) decreased in rats pretreated with PA-MSHA every other day for 16 days. The survival rate of rats pretreated with 0.5ml PA-MSHA for 8 days (91.7%) was higher than that of rats pretreated for 16 days (41.7%) and that of rats without pretreatment (33.3%). The results are related to the regulation of inflammatory mediators and the induction of hyporeactivity to endotoxin by PA-MSHA. Binding Gram-negative bacterial endotoxin (such as LPS) to TLR-4 triggers a signal transduction cascade. Similar to LPS, PA-MSHA can also activate immune response through signal transduction mediated by Toll-like receptors, and endotoxin tolerance can be induced by PA-MSHA pretreatment. Endotoxin tolerance may have a protective effect in the early stage of sepsis. However, long-term tolerance may lead to immunosuppression in the late stage of sepsis and increase the risk of nosocomial infection and subsequent death. This study suggests that accurate control of the dose and time of PA-MSHA administration can effectively improve the survival rate of CLP rats. In this study, the patients in the treatment group showed an increased inflammatory response, similar to the level of pro-inflammatory factors (TNF- α and IL-6) in the 8-day pretreated mice such as Zhu, H (55). It can be speculated that PA-MSHA may play a specific role in preventing and treating sepsis. However, it is only a preliminary study, and its mechanism and value need to be further studied.

#### 4.3.2 CD4+CD8+ double-positive T cell

Immunotherapy methods emerge endlessly with the in-depth study of immune mechanisms, but most still need to be mature. In this study, it was found that PA-MSHA injection had a positive effect on the immunity of patients before the operation. After treatment with PA-MSHA before PCNL, the number of peripheral CD4+CD8+DPT cells in the treatment group was higher than that in the control group, and the difference was statistically significant. With peripheral CD4+CD8+DPT cells as protective immune cells, it can be inferred that PA-MSHA has an immunomodulatory and protective effect on PCNL patients with infection.

CD4+CD8+ double-positive T cells (CD4+CD8+double positive T cells, CD4+CD8+DPT) were previously widely believed to be a crucial stage in developing T cells in the thymus and differentiate into CD4+ or CD8+ cells mediated by transcription factors ThPOK or Runx3 (56). At present, little is known about the function, function, and biological significance of peripheral CD4+CD8+DPT cells. In recent years, it has been found that CD4+CD8+DPT cells found in blood and peripheral lymphoid tissue, CD4+T cells, CD8+T cells, and thymus may be its sources, as mature subsets and conventional CD4+T cells and CD8+T cells and exist in the periphery (57, 58). Through single-cell RNA sequencing, scholars found that in terms of functional gene expression, circulating CD4+CD8+DPT cells had not only the characteristics of immature cells but also had the characteristics of helper cells, regulatory cells, cytotoxic cells, and congenital-like cells, showing more functions than single CD4+ and CD8+T cells (58). In response to Mycobacterium tuberculosis infection, peripheral CD4+CD8+DPT cells have both pro-inflammatory and anti-inflammatory effects, expressing more cytolytic markers (CD107a, granzyme B, and perforin), Th1 type cytokines (TNF, IL-2, IFN- γ), Th17 type cytokines, IL-10 (59). It has been found that peripheral CD4+CD8+DPT cells are likely to play a role in protective immunity in animals (60–62). In addition, peripheral CD4+CD8+DPT cells express immune memory markers, which can produce rapid Th1 response and cytotoxic response to previously infected or highly replicated persistent infected viruses, antigens, and vaccines (58, 63). In patients with acquired immunodeficiency syndrome (acquired immunodeficiency syndrome, AIDS), it has been found that CD4+CD8+DPT cells can make multiple immune responses to HIV antigens to suppress HIV, which is an essential part of AIDS-specific cellular immune response (64). The DPT cells shared the similar characteristics with cytotoxic T cells (CTL) and exerted an anti-viral role in HFRS (65).

It is also found that CD4+CD8+DPT cells may play a vital role in the production of autoantibodies in systemic lupus erythematosus (66). The proportion of DPT cells could be a potential marker to evaluate Lupus nephritis susceptibility (67). However, its negative effects were found in some studies. One study found that reveals human DPTs as a T cell population directly involved with graft-versus-host disease (GVHD) pathology (68). Expansion of DPT cells is associated with joint damage and frequent escalation of therapy, possibly suggesting a contribution to more severe rheumatoid arthritis (69).

### 4.4 Changes of immune function before and after PCNL

People with immune system defects are vulnerable to severe and even life-threatening infections, which fully illustrates the importance of a complete immune system to overall health. The causes of immunodeficiency can be divided into congenital and acquired. Congenital immunodeficiency mainly refers to the developmental dysfunction of T cells and B cells, acquired immunodeficiencies include HIV infection, iatrogenic (post-organ transplant) immunosuppression, surgery, and trauma (70), and anesthetic medications may affect the immune response by regulating neurohumoral responses to immunoreactive cells (71). The analgesic medications sufentanil and remifentanil used in this study belong to opioids. One of the potential side effects of opioids is that they affect the immune system. Some studies have shown that opioids can induce the immunosuppression of the adaptive immune system and innate immune system and the destruction of the mucosal barrier. However, its effect is more evident in animals (72). Anesthesia, pain, surgical stress, tissue damage, blood transfusions, and invasive microorganisms can stimulate patients to produce complex immune responses that increase the susceptibility to postoperative infection (71, 73). The degree of lymphocyte inhibition is related to the complexity of the operation or the severity of the injury. The degree of lymphocyte inhibition was associated with subsequent infection complications and mortality (74).

In this study, the percentage of CD3+T cells, immunoglobulin, and complement decreased in the control group after the operation; on the contrary, the percentage of NK cells and NKT cells increased, which was similar to the immune changes observed during subarachnoid surgery, hemorrhage, and cardiac surgery (13, 75). It is suggested that anesthesia and surgical injury may lead to the activation of the innate immune system and the inhibition of the acquired immune system. Albertsmeier M. et al. (76) also proposed that in the early stage after traumatic surgery, although antigen-presenting cells were continuously activated, the function of T lymphocytes was suppressed. In the treatment group, CD4+CD8+DPT cells increased, immunoglobulin and complement decreased, and no similar immune changes were observed in CD3+T cells, NK cells, and NKT cells. However, there was no statistical difference between the two groups after the operation, so it can not be considered the effect of PA-MSHA medications.

### 4.5 Limitations and deficiencies of research

This study’s main limitation lies in its retrospective study design of a single institution, which may have potential bias. Sepsis is one of the outcome indicators, but its incidence is low, and an ideal result has not been obtained. More clinical samples and data need to be collected in later studies to find more valuable conclusions.

PA-MSHA injection belongs to the type of vaccine which plays a role by stimulating the immune response. However, its mechanism and the choice of treatment time still need to be further studied.

## 5 Conclusion

This study found that patients with upper urinary tract calculi are complicated with infection treated with PA-MSHA based on antibiotics before percutaneous nephrolithotomy, which may play a specific role in preventing and treating sepsis. However, its mechanism and mechanism and value need to be further studied. The percentage of double-positive T cells in peripheral blood increased after PA-MSHA treatment, which may have an immunomodulatory and protective effect on PCNL patients with calculi complicated with infection. PCNL surgery and anesthetic trauma may inhibit some of the patients’ cellular and humoral immune functions.

## Data availability statement

The original contributions presented in the study are included in the article/supplementary material. Further inquiries can be directed to the corresponding authors.

## Ethics statement

The studies involving human participants were reviewed and approved by the ethics committee of The Second Affiliated Hospital of Kunming Medical University. Written informed consent was obtained from the [individual(s) AND/OR minor(s)’ legal guardian/next of kin] for the publication of any potentially identifiable images or data included in this article.

## Author contributions

JL contributed to the conception of the study. HL helped for funding acquisition,and supervision. YZha contributed significantly to analysis and manuscript preparation. YaZ helped perform the analysis. RZ helped for visualization and investigation. JZ, GZ and YZho both helped for Supervision. All authors contributed to the article and approved the submitted version.

## References

[1] Rudd KE Johnson SC Agesa KM Shackelford KA Tsoi D Kievlan DR. Global, regional, and national sepsis incidence and mortality, 1990-2017: analysis for the global burden of disease study. Lancet (London England) (2020) 395(10219):200–11. doi: 10.1016/S0140-6736(19)32989-7 PMC6970225 31954465

[2] Finkelsztein EJ Jones DS Ma KC Pabón MA Delgado T Nakahira K. Comparison of qSOFA and SIRS for predicting adverse outcomes of patients with suspicion of sepsis outside the intensive care unit. Crit Care (London England) (2017) 21(1):73. doi: 10.1186/s13054-017-1658-5 PMC5366240 28342442

[3] Chen D Jiang C Liang X Zhong F Huang J Lin Y. Early and rapid prediction of postoperative infections following percutaneous nephrolithotomy in patients with complex kidney stones. BJU Int (2019) 123(6):1041–7. doi: 10.1111/bju.14484 30007112

[4] Wollin DA Joyce AD Gupta M Wong MYC Laguna P Gravas S. Antibiotic use and the prevention and management of infectious complications in stone disease. World J Urol (2017) 35(9):1369–79. doi: 10.1007/s00345-017-2005-9 28160088

[5] Rivera M Viers B Cockerill P Agarwal D Mehta R Krambeck A. Pre- and postoperative predictors of infection-related complications in patients undergoing percutaneous nephrolithotomy. J Endourol (2016) 30(9):982–6. doi: 10.1089/end.2016.0191 27393153

[6] Boonen E Van den Berghe G. Understanding the HPA response to critical illness: novel insights with clinical implications. Intensive Care Med (2015) 41(1):131–3. doi: 10.1007/s00134-014-3545-8 25406407

[7] Venet F Monneret G. Advances in the understanding and treatment of sepsis-induced immunosuppression. Nat Rev Nephrol (2017) 14(2):121–37. doi: 10.1038/nrneph.2017.165 29225343

[8] Opal SM Cross AS Bhattacharjee AK Visvanathan K Zabriskie JB. Immunoprophylaxis against bacterial sepsis. Sepsis (1999) 3(3):225–34. doi: 10.1023/A:1009804003941

[9] Nedeva C. Inflammation and cell death of the innate and adaptive immune system during sepsis. Biomolecules (2021) 11(7). doi: 10.3390/biom11071011 PMC8301842 34356636

[10] Wei Y Liu D Jin X Gao P Wang Q Zhang J. PA-MSHA inhibits the growth of doxorubicin-resistant MCF-7/ADR human breast cancer cells by downregulating Nrf2/p62. Cancer Med (2016) 5(12):3520–31. doi: 10.1002/cam4.938 PMC5224842 27758045

[11] Zhou R Xu J He J Gong Y Wang H Linghu H. Topical application of pseudomonas aeruginosa-mannose sensitive hemagglutinin (PA-MSHA) for refractory lymphatic leakage following lymphadenectomy in patients with gynecological malignancies. Cancer Manage Res (2021) 13:4873–8. doi: 10.2147/CMAR.S307700 PMC8232861 34188540

[12] Xiu D Cheng M Zhang W Ma X Liu L. Pseudomonas aeruginosa-mannose-sensitive hemagglutinin inhibits chemical-induced skin cancer through suppressing hedgehog signaling. Exp Biol Med (Maywood NJ) (2020) 245(3):213–20. doi: 10.1177/1535370219897240 PMC7045328 31903775

[13] Zhou Y Jiang Y Peng Y Zhang M. The quantitative and functional changes of postoperative peripheral blood immune cell subsets relate to prognosis of patients with subarachnoid hemorrhage: a preliminary study. World Neurosurg (2017) 108:206–15. doi: 10.1016/j.wneu.2017.08.091 28866066

[14] Porat A Bhutta BS Kesler S. Urosepsis. StatPearls. Treasure Island (FL: StatPearls Publishing Copyright © 2022, StatPearls Publishing LLC (2022).

[15] Singer M Deutschman CS Seymour CW Shankar-Hari M Annane D Bauer M. The third international consensus definitions for sepsis and septic shock (Sepsis-3). Jama (2016) 315(8):801–10. doi: 10.1001/jama.2016.0287 PMC4968574 26903338

[16] Dreger NM Degener S Ahmad-Nejad P Wöbker G Roth S. Urosepsis–etiology, diagnosis, and treatment. Deutsches Arzteblatt Int (2015) 112(49):837–47. doi: 10.3238/arztebl.2015.0837 PMC4711296 26754121

[17] Bonkat G Cai T Veeratterapillay R Bruyère F Bartoletti R Pilatz A. Management of urosepsis in 2018. Eur Urol focus (2019) 5(1):5–9. doi: 10.1016/j.euf.2018.11.003 30448051

[18] Hotchkiss RS Monneret G Payen D. Sepsis-induced immunosuppression: from cellular dysfunctions to immunotherapy. Nat Rev Immunol (2013) 13(12):862–74. doi: 10.1038/nri3552 PMC4077177 24232462

[19] Venet F Demaret J Gossez M Monneret G. Myeloid cells in sepsis-acquired immunodeficiency. Ann New York Acad Sci (2021) 1499(1):3–17. doi: 10.1111/nyas.14333 32202669

[20] Patil NK Guo Y Luan L Sherwood ER. Targeting immune cell checkpoints during sepsis. Int J Mol Sci (2017) 18(11):2413. doi: 10.3390/ijms18112413 29135922 PMC5713381

[21] Voll RE Herrmann M Roth EA Stach C Kalden JR Girkontaite I. Immunosuppressive effects of apoptotic cells. Nature (1997) 390(6658):350–1. doi: 10.1038/37022 9389474

[22] Biswas SK Lopez-Collazo E. Endotoxin tolerance: new mechanisms, molecules and clinical significance. Trends Immunol (2009) 30(10):475–87. doi: 10.1016/j.it.2009.07.009 19781994

[23] Andreu-Ballester JC Tormo-Calandín C Garcia-Ballesteros C Pérez-Griera J Amigó V Almela-Quilis A. Association of γδ T cells with disease severity and mortality in septic patients. Clin Vaccine Immunol CVI (2013) 20(5):738–46. doi: 10.1128/CVI.00752-12 PMC3647746 23515014

[24] Hotchkiss RS Tinsley KW Swanson PE Grayson MH Osborne DF Wagner TH. Depletion of dendritic cells, but not macrophages, in patients with sepsis. J Immunol (Baltimore Md 1950) (2002) 168(5):2493–500. doi: 10.4049/jimmunol.168.5.2493 11859143

[25] Faivre V Lukaszewicz AC Alves A Charron D Payen D Haziot A. Human monocytes differentiate into dendritic cells subsets that induce anergic and regulatory T cells in sepsis. PloS One (2012) 7(10):e47209. doi: 10.1371/journal.pone.0047209 23071758 PMC3468528

[26] Gautier EL Huby T Saint-Charles F Ouzilleau B Chapman MJ Lesnik P. Enhanced dendritic cell survival attenuates lipopolysaccharide-induced immunosuppression and increases resistance to lethal endotoxic shock. J Immunol (Baltimore Md 1950) (2008) 180(10):6941–6. doi: 10.4049/jimmunol.180.10.6941 18453615

[27] Cook CH Trgovcich J. Cytomegalovirus reactivation in critically ill immunocompetent hosts: a decade of progress and remaining challenges. Antiviral Res (2011) 90(3):151–9. doi: 10.1016/j.antiviral.2011.03.179 PMC3129598 21439328

[28] Kovach MA Standiford TJ. The function of neutrophils in sepsis. Curr Opin Infect diseases (2012) 25(3):321–7. doi: 10.1097/QCO.0b013e3283528c9b 22421753

[29] Pachot A Monneret G Voirin N Leissner P Venet F Bohé J. Longitudinal study of cytokine and immune transcription factor mRNA expression in septic shock. Clin Immunol (Orlando Fla) (2005) 114(1):61–9. doi: 10.1016/j.clim.2004.08.015 15596410

[30] Guignant C Lepape A Huang X Kherouf H Denis L Poitevin F. Programmed death-1 levels correlate with increased mortality, nosocomial infection and immune dysfunctions in septic shock patients. Crit Care (London England) (2011) 15(2):R99. doi: 10.1186/cc10112 PMC3219369 21418617

[31] Venet F Chung CS Kherouf H Geeraert A Malcus C Poitevin F. Increased circulating regulatory T cells (CD4(+)CD25 (+)CD127 (-)) contribute to lymphocyte anergy in septic shock patients. Intensive Care Med (2009) 35(4):678–86. doi: 10.1007/s00134-008-1337-8 PMC2789433 18946659

[32] Venet F Pachot A Debard AL Bohe J Bienvenu J Lepape A. Human CD4+CD25+ regulatory T lymphocytes inhibit lipopolysaccharide-induced monocyte survival through a Fas/Fas ligand-dependent mechanism. J Immunol (Baltimore Md 1950) (2006) 177(9):6540–7. doi: 10.4049/jimmunol.177.9.6540 17056586

[33] Alejandria MM Lansang MA Dans LF Mantaring JB 3rd. Intravenous immunoglobulin for treating sepsis, severe sepsis and septic shock. Cochrane Database systematic Rev (2013) 2013(9):Cd001090. doi: 10.1002/14651858.CD001090.pub2 PMC6516813 24043371

[34] Brubaker SW Brewer SM Massis LM Napier BA Monack DM. A rapid caspase-11 response induced by IFNγ priming is independent of guanylate binding proteins. iScience (2020) 23(10):101612. doi: 10.1016/j.isci.2020.101612 33089101 PMC7566093

[35] Unsinger J McGlynn M Kasten KR Hoekzema AS Watanabe E Muenzer JT. IL-7 promotes T cell viability, trafficking, and functionality and improves survival in sepsis. J Immunol (Baltimore Md 1950) (2010) 184(7):3768–79. doi: 10.4049/jimmunol.0903151 PMC2914630 20200277

[36] Feng Z Shi Q Fan Y Wang Q Yin W. Ulinastatin and/or thymosin α1 for severe sepsis: a systematic review and meta-analysis. J Trauma Acute Care Surg (2016) 80(2):335–40. doi: 10.1097/TA.0000000000000909 26517783

[37] Nakamori Y Park EJ Shimaoka M. Immune deregulation in sepsis and septic shock: reversing immune paralysis by targeting PD-1/PD-L1 pathway. Front Immunol (2020) 11:624279. doi: 10.3389/fimmu.2020.624279 33679715 PMC7925640

[38] Opal SM Palardy JE Chen WH Parejo NA Bhattacharjee AK Cross AS. Active immunization with a detoxified endotoxin vaccine protects against lethal polymicrobial sepsis. J Infect Dis (2005) 192(6):2074–80. doi: 10.1086/498167 16288370

[39] Ioanna Z Katerina B Irene A. Immunotherapy-on-Chip against an experimental sepsis model. Inflammation (2021) 44(6):2333–45. doi: 10.1007/s10753-021-01506-y 34417666

[40] Zhao J Wang M Yang Y Wang G Che F Li Q. CD123 thioaptamer protects against sepsis *via* the blockade between IL-3/CD123 in a cecal ligation and puncture rat model. Nucleosides nucleotides Nucleic Acids (2021) 40(1):16–31. doi: 10.1080/15257770.2020.1815770 32985358

[41] Saito M Inoue S Yamashita K Kakeji Y Fukumoto T Kotani J. IL-15 improves aging-induced persistent T cell exhaustion in mouse models of repeated sepsis. Shock (Augusta Ga) (2020) 53(2):228–35. doi: 10.1097/SHK.0000000000001352 31935201

[42] Troia R Mascalzoni G Agnoli C Lalonde-Paul D Giunti M Goggs R. Cytokine and chemokine profiling in cats with sepsis and septic shock. Front veterinary science (2020) 7:305. doi: 10.3389/fvets.2020.00305 PMC7273843 32548135

[43] Li T Yang L Fu S-J Xiao E-L Yuan X Lu J-Z. Subcutaneous injections of the mannose-sensitive hemagglutination pilus strain of pseudomonas aeruginosa stimulate host immunity, reduce bladder cancer size and improve tumor survival in mice. Cell Biochem Biophysics (2015) 73(1):245–52. doi: 10.1007/s12013-015-0611-y 25724441

[44] Jian X Chao S Xiaoli Z Aiwu W. Inactivated p. aeruginosa restores immune imbalance of chronic idiopathic urticaria. Arch Dermatol Res (2020) 312(5):353–9. doi: 10.1007/s00403-019-02019-3 31797034

[45] Chang L Xiao W Yang Y Li H Xia D Yu G. Pseudomonas aeruginosa-mannose-sensitive hemagglutinin inhibits epidermal growth factor receptor signaling pathway activation and induces apoptosis in bladder cancer cells *in vitro* and in vivo. Urologic Oncol (2014) 32(1):36.e11–8. doi: 10.1016/j.urolonc.2013.02.013 23948182

[46] Lv F Cao J Liu Z Wang Z Zhang J Zhang S. Phase II study of pseudomonas aeruginosa-Mannose-Sensitive hemagglutinin in combination with capecitabine for her-2-negative metastatic breast cancer pretreated with anthracycline and taxane. PloS One (2015) 10(3):e0118607. doi: 10.1371/journal.pone.0118607 25768439 PMC4359133

[47] Zhao X Wu X Yu W Cai X Liu Q Fu X. PA-MSHA inhibits proliferation and induces apoptosis in human non-small cell lung cancer cell lines with different genotypes. Mol Med Rep (2016) 14(6):5369–76. doi: 10.3892/mmr.2016.5869 27779712

[48] Liu J Duan X. PA-MSHA induces apoptosis and suppresses metastasis by tumor associated macrophages in bladder cancer cells. Cancer Cell Int (2017) 17:76. doi: 10.1186/s12935-017-0445-3 28824336 PMC5561576

[49] Zhang C Zhang Z Wang L Han J Li F Shen C. Pseudomonas aeruginosa-mannose sensitive hemagglutinin injection treated cytokine-induced killer cells combined with chemotherapy in the treatment of malignancies. Int Immunopharmacol (2017) 51:57–65. doi: 10.1016/j.intimp.2017.08.003 28802902

[50] Liu XF Wang L Qu Y Zhong DW Miao XY Yao HL. Effect of the PA-MSHA vaccine on septic serum-induced inflammatory response. Mol Med Rep (2013) 7(4):1350–4. doi: 10.3892/mmr.2013.1337 23440442

[51] Yuan D Li A. Value of PCT, IL-6 and CRP in diagnosis of sepsis. China J Modern Med (2018) 28(32):86–90.

[52] Mengli Z Liqing P Guangxin Y Hong P Ying C Zhi-chao W. Prognostic value of SIRS criteria,SOFA and qSOFA scores in patients with suspected infection in emergency department. Chin J Nosocomiol (2019) 29(19):2894–8.

[53] Singh P Yadav S Singh A Saini AK Kumar R Seth A. Systemic inflammatory response syndrome following percutaneous nephrolithotomy: assessment of risk factors and their impact on patient outcomes. Urologia Internationalis (2016) 96(2):207–11. doi: 10.1159/000441954 26745881

[54] Peng Y Zhang W Xu Y Li L Yu W Zeng J. Performance of SOFA, qSOFA and SIRS to predict septic shock after percutaneous nephrolithotomy. World J Urol (2021) 39(2):501–10. doi: 10.1007/s00345-020-03183-2 32277278

[55] Zhu H Wang S Shen L Wang W Zhao F Cao T. Effects of pseudomonas aeruginosa mannose-sensitive hemagglutinin (PA-MSHA) pretreatment on septic rats. Int Immunopharmacol (2013) 17(3):836–42. doi: 10.1016/j.intimp.2013.09.006 24055021

[56] Overgaard NH Jung JW Steptoe RJ Wells JW. CD4+/CD8+ double-positive T cells: more than just a developmental stage? J leukocyte Biol (2015) 97(1):31–8. doi: 10.1189/jlb.1RU0814-382 25360000

[57] Van Kaer L Rabacal WA Scott Algood HM Parekh VV Olivares-Villagómez D. *In vitro* Induction of regulatory CD4+CD8α+ T cells by TGF-β, IL-7 and IFN-γ. PloS One (2013) 8(7):e67821. doi: 10.1371/journal.pone.0067821 23844100 PMC3701067

[58] Choi SM Park HJ Choi EA Jung KC Lee JI. Cellular heterogeneity of circulating CD4(+)CD8(+) double-positive T cells characterized by single-cell RNA sequencing. Sci Rep (2021) 11(1):23607. doi: 10.1038/s41598-021-03013-4 34880348 PMC8655006

[59] Diedrich CR Gideon HP Rutledge T Baranowski TM Maiello P Myers AJ. CD4CD8 double positive T cell responses during mycobacterium tuberculosis infection in cynomolgus macaques. J Med Primatol (2019) 48(2):82–9. doi: 10.1111/jmp.12399 PMC6519377 30723927

[60] Nam K Akari H Terao K Shibata H Kawamura S Yoshikawa Y. Peripheral blood extrathymic CD4(+)CD8(+) T cells with high cytotoxic activity are from the same lineage as CD4(+)CD8(-) T cells in cynomolgus monkeys. Int Immunol (2000) 12(7):1095–103. doi: 10.1093/intimm/12.7.1095 10882421

[61] Zuckermann FA. Extrathymic CD4/CD8 double positive T cells. Veterinary Immunol Immunopathol (1999) 72(1-2):55–66. doi: 10.1016/S0165-2427(99)00118-X 10614493

[62] Redant V Favoreel HW Dallmeier K Van Campe W De Regge N. Japanese Encephalitis virus persistence in porcine tonsils is associated with a weak induction of the innate immune response, an absence of IFNγ mRNA expression, and a decreased frequency of CD4(+)CD8(+) double-positive T cells. Front Cell infection Microbiol (2022) 12:834888. doi: 10.3389/fcimb.2022.834888 PMC8908958 35281443

[63] Nascimbeni M Shin EC Chiriboga L Kleiner DE Rehermann B. Peripheral CD4(+)CD8(+) T cells are differentiated effector memory cells with antiviral functions. Blood (2004) 104(2):478–86. doi: 10.1182/blood-2003-12-4395 15044252

[64] Frahm MA Picking RA Kuruc JD McGee KS Gay CL Eron JJ. CD4+CD8+ T cells represent a significant portion of the anti-HIV T cell response to acute HIV infection. J Immunol (Baltimore Md 1950) (2012) 188(9):4289–96. doi: 10.4049/jimmunol.1103701 PMC3692005 22461689

[65] Zhang H Wang Y Ma Y Tang K Zhang C Wang M. Increased CD4(+)CD8(+) double positive T cells during hantaan virus infection. Viruses (2022) 14(10):2243. doi: 10.3390/v14102243 36298798 PMC9611689

[66] Wu Y Cai B Feng W Yang B Huang Z Zuo C. Double positive CD4+CD8+ T cells: key suppressive role in the production of autoantibodies in systemic lupus erythematosus. Indian J Med Res (2014) 140(4):513–9. PMC4277137 25488445

[67] Chang K Na W Liu C Xu H Liu Y Wang Y. Peripheral CD4 (+)CD8 (+) double positive T cells: a potential marker to evaluate renal impairment susceptibility during systemic lupus erythematosus. J Biomed Res (2022) 37(1):59–68. doi: 10.7555/JBR.36.20220094 36625011 PMC9898043

[68] Hess NJ Turicek DP Riendeau J McIlwain SJ Contreras Guzman E Nadiminti K. Inflammatory CD4/CD8 double-positive human T cells arise from reactive CD8 T cells and are sufficient to mediate GVHD pathology. Sci Adv (2023) 9(12):eadf0567. doi: 10.1126/sciadv.adf0567 36961891 PMC10038349

[69] Nguyen P Melzer M Beck F Krasselt M Seifert O Pierer M. Expansion of CD4+CD8+ double-positive T cells in rheumatoid arthritis patients is associated with erosive disease. Rheumatol (Oxford England) (2022) 61(3):1282–7. doi: 10.1093/rheumatology/keab551 34260705

[70] Sjaastad FV Kucaba TA Dileepan T Swanson W Dail C Cabrera-Perez J. Polymicrobial sepsis impairs antigen-specific memory CD4 T cell-mediated immunity. Front Immunol (2020) 11:1786. doi: 10.3389/fimmu.2020.01786 32903436 PMC7435018

[71] von Dossow V Sander M MacGill M Spies C. Perioperative cell-mediated immune response. Front bioscience J virtual library (2008) 13:3676–84. doi: 10.2741/2958 18508464

[72] Plein LM Rittner HL. Opioids and the immune system - friend or foe. Br J Pharmacol (2018) 175(14):2717–25. doi: 10.1111/bph.13750 PMC6016673 28213891

[73] Yongguang X Bo W Zhifu M. Relation between surgery and immune function in patients with lung cancer. J Chin Oncol (2019) 25(03):206–8.

[74] Kimura F Shimizu H Yoshidome H Ohtsuka M Miyazaki M. Immunosuppression following surgical and traumatic injury. Surg Today (2010) 40(9):793–808. doi: 10.1007/s00595-010-4323-z 20740341 PMC7101797

[75] Hauser GJ Chan MM Casey WF Midgley FM Holbrook PR. Immune dysfunction in children after corrective surgery for congenital heart disease. Crit Care Med (1991) 19(7):874–81. doi: 10.1097/00003246-199107000-00009 2055075

[76] Albertsmeier M Quaiser D von Dossow-Hanfstingl V Winter H Faist E Angele MK. Major surgical trauma differentially affects T-cells and APC. Innate Immunity (2015) 21(1):55–64. doi: 10.1177/1753425913516659 24398860

